# Association of Notch-1, osteopontin and stem-like cells in ENU-glioma malignant process

**DOI:** 10.18632/oncotarget.25808

**Published:** 2018-07-31

**Authors:** Susana Bulnes, Garazi Bermúdez, José Vicente Lafuente

**Affiliations:** ^1^ LaNCE, Department of Neuroscience, University of the Basque Country (UPV/EHU), Leioa, Spain; ^2^ Neurosurgery Service, Cruces University Hospital, Barakaldo, Spain; ^3^ Nanoneurosurgery Group, Institute of Health Research Biocruces, Barakaldo, Spain; ^4^ Faculty of Health Science, Universidad Autónoma de Chile, Santiago de Chile, Chile

**Keywords:** angiogenesis, glioma stem-like cells, N-ethyl-N-nitrosourea, Notch-1, osteopontin

## Abstract

Notch-1 and osteopontin (OPN) mediate angiogenesis and glioma stem-like cell (GSLC) maintenance. However, the relationship between these molecules and GSLCs during the development of glioma is unknown. We investigate the expression of Notch-1, OPN and vascular endothelial growth factor (VEGF) associated to the stemness markers nestin and CD133 in three stages of murine gliomas induced by N-ethyl-N-nitrosourea (ENU).

Notch-1 and OPN overexpress in the intermediate stage (II), which corresponds to the “angiogenesis switch”. Nestin+ cells appear in all stages of ENU-glioma but CD133 only from stage II on. In stage III, neoplastic cells expressing nestin, CD133 and nestin/CD133 reside in spheroid-like aggregates (SAs) and in the neoangiogenic border. These aggregates show Notch-1 and VEGF+ surrounding cells and a significant size and density increase with respect to stage I (3.3 ± 1.5 to 22.4 ± 6.3 µm^2^, n° = 0.3 ± 0.1 to 4.2 ± 0.9, from stage I to stage III, respectively).

OPN expression increases in correlation to the glioma malignancy from 4.5 ± 1.8% (I) to 12.3 ± 1.2% of OPN+ cells (III). It predominates in astrocyte-like cells of the neoangiogenic border, displaying co-location with VEGF and CD133. The OPN immunopositivity distribution correlates with the CD133 distribution.

In conclusion, OPN co-expressing with CD133 contributes to the identification of GSLCs in the neoangiogenic border, while Notch-1 is present around SAs in advanced stages. The ENU-glioma, mainly in stage II, is a useful tool for assessing new antitumour therapies against these molecules.

## INTRODUCTION

Glioblastoma (GBM) is the most malignant solid tumor of the central nervous system (CNS), with poor prognosis due to a high proliferative and invasive capacity, besides resistance to current therapies [1]. The growth of GBM is angiogenesis-dependent, therefore several antitumor therapies are focused against this process, trying to interfere with molecules that participate in this process, such as hypoxia inducible factor 1 alpha (HIF-1alpha), vascular endothelial growth factor (VEGF) or its receptors (VEGFR2, VEGFR1), erythropoietin (EPO) and endothelial nitric oxide synthase (eNOS) [2, 3]. Gliomas develop resistance to antiangiogenic molecules by mechanisms frequently involving cancer stem cells (CSCs) [4, 5].

Glioma stem-like cells (GSLCs) represent a small subpopulation of cells showing stemness-associated properties and playing a pivotal role in angiogenesis [6, 7]. These cells, in addition to the capacity for self-renewal and multilineage differentiation [8], are responsible for radio-chemo-resistance and subsequent tumor recurrence [9]. Osteopontin (OPN) and Notch-1 are two molecules proposed as being involved in mediating this resistance [10–12].

Notch signalling is an intercellular signalling pathway that plays an important role in reprogramming neoplastic cells towards the GSLC phenotype [13, 14]. To date, four Notch receptors (Notch 1-4) with five corresponding ligands namely Delta-like-1, Delta-like-3, Delta-like-4, Jagged-1 and Jagged-2, have been identified in humans. Notch-1 is a transmembrane receptor involved in cell-cell signalling that has been related to glioma survival and proliferation [15].

OPN has been linked to tumor progression, angiogenesis and metastasis formation [16]. It is a member of the Small Integrin-Binding Ligand N-linked Glycoproteins (SIBLINGs). They are a family of extracellular matrix proteins involved in the acquisition/maintenance of stemness characteristics and tumorigenicity of GSLCs [17]. Its overexpression is shown in hypoxia and has been related to a poorer prognosis in GBM [18].

Consequently, GSLCs and molecules related to their maintenance are currently considered a potential therapeutic target for antitumor therapies [19]. Despite this, the cellular heterogeneity of GBM and the lack of specific markers make it difficult to identify GSLCs. GSLCs from GBM exhibit different phenotypes, with increasing interest in the potential significance of cancer stem cells with respect to diagnosis, prognosis and development of novel therapeutic targets [20].

Several authors use markers of stemness (SOX2, OLIG4, CD15, nestin or CD133) to identify dedifferentiated neoplastic cells [21–25]. CD133 (a membrane marker also known as Prominin-1) and nestin (a type IV intermediate filament protein) are two immunomarkers widely associated with stemness phenotype acquisition mediated by hypoxia [26]. The microenvironment plays a pivotal role in inducing cell dedifferentiation and the stemness phenotype. Wang *et al.* [27] have shown the role played by hypoxia in cell dedifferentiation. They marked cells *in vitro* by CD133-CD15-Nestin and demonstrated via *in vivo* assays the tumorigenic capacity of these selected cells under hypoxia conditions. Nestin and CD133 have been associated with GSLCs located in perivascular niches of tumour microvessels [28].

In previous work, we have studied the angiogenesis process in the ENU-glioma model [2, 29, 30]. ENU is a nitrosourea that after prenatal exposure induces glial tumours in the central nervous system. It acts by alkylating O6-guanine, O2-thymine and O4-thymine, inducing mutations of certain oncogenes such as p53 and genes coding for caspase-9, platelet-derived growth factor receptor alpha (PDGFRα), CDKN2A and EGFR, all related to the genesis of glial tumours [31, 32]. Therefore, this model reproduces quite closely the natural development and neuropathology of human gliomas [30, 33].

We described an overexpression of VEGF in the intermediate stage of ENU-glioma [30]. This stage, which corresponded to the “angiogenesis switch”, was characterised by an increase of microvascular density and an increase of VEGF+ cells in the border areas and around the aberrant microvessels [33]. Nestin as well as CD133 were expressed in cells located in areas showing features of hypoxia and associated with aberrant microvessels, conforming clusters called spheroid-like aggregates (SAs) [29, 34].

Therefore, in this work, since the Notch-1 and OPN molecules are related to the maintenance of angiogenesis and GSLCs, we analyze the distribution of Notch-1 and OPN immunopositivity in relation to nestin and CD133 and the proangiogenic factor VEGF in early to advanced stages of ENU-gliomas.

## RESULTS

### Expression of Nestin and CD133 markers in different stages of ENU-glioma

**Figure 1:**
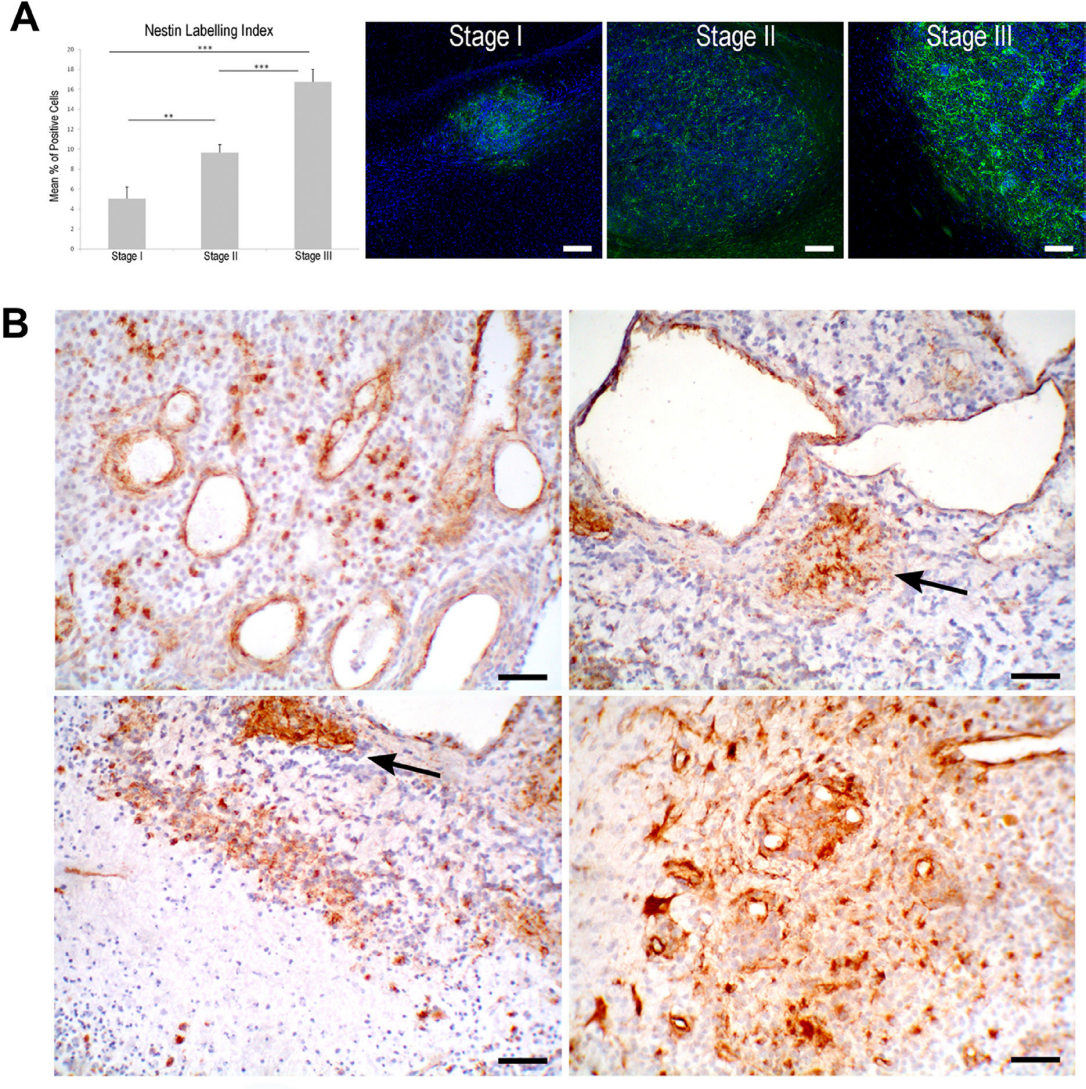
Nestin expression in ENU-glioma development (**A**) Statistical increase of nestin+ cell density during ENU-glioma progression, shown graphically. ^*^p < 0.05, ^**^p < 0.01, ^***^p < 0.001. (**B**) In stage III, there are nestin+ cells isolated throughout the neoplasia or distributed in clusters termed spheroid-like aggregates (SAs, arrow). These clusters are located in the perivascular area of huge dilated microvessels. Aggregations of nestin+ cells are also shown in hypoxic areas of pseudopalisading necrosis and in the periphery of the tumor, associated with glomeruloid vessels. Scale bar of 50 μm.

81 gliomas from 53 rats are segregated into three stages of malignancy (*n =* 27, Table 1) according to parameters described in our previous works [30, 33, 34]. Stage I corresponds to low-grade glioma. It represents small masses of proliferating cells with isomorphic morphology that develop mainly inside subcortical white matter. Few nestin+ cells are found distributed throughout these masses (nestin-LI of 4.8 ± 0.57) (Figure 1A). Stage II corresponds to nodules showing anaplastic changes and increase of nestin+ cells (nestin-LI of 9.69 ± 0.84). Labelled cells appear either inside the tumour or in the border area, building aggregates around the microvessels or isolated called SAs. (Figure 1B). Nestin+ cells show two different morphologies, small round cells similar to stem cells and large cells with elongated processes. Stage III is the advanced anaplastic glioma corresponding to glioblastoma (GBM). This stage shows the highest density of nestin+ cells (nestin-LI of 16.67 ± 1.36) (Figure 1A) and a clear pattern of distribution in the border area of the tumour.

**Table 1:** Characteristics of the three stages of ENU-glioma

Parameters of ENU-glioma classification		Stage I	Stage II	Stage III
**Tumour size (mm**^2^**)**	**Range** **Mean**	0.5 to 1.9 1.2 ± 0.3	2.7 to 4.9 3.8 ± 0.5	7.3 to 14.3 10.7 ± 1.6
**Proliferation index (KI-67 PI, % of cells)**	**Range** **Mean**	2.4 to 6.2 4.2 ± 0.5	6.04 to 12.8 9.35 ± 0.8	11.6 to 28 16.8 ± 1.9

Parameters to take into account to segregate the experimental gliomas induced by a single dose of N-ethyl-N-nitrosourea to prenatal Sprague Dawley rats.

Abbreviations: KI-67 PI: proliferation index.

Immunoexpression for CD133 is found in small round-shaped cells since stage II and it follows the same distribution pattern as nestin. In stage III, nestin+, CD133+ and nestin/CD133+ cells are found aggregated into SAs distributed thorough the neoplasia, in perinecrotic areas or close to aberrant microvessels. Tumor border area shows plenty of these cells located near the glomeruloid vessels and delimiting the periphery area of the neoplasia (Figure 1B).

### Spheroid-like aggregates associated cells

**Figure 2:**
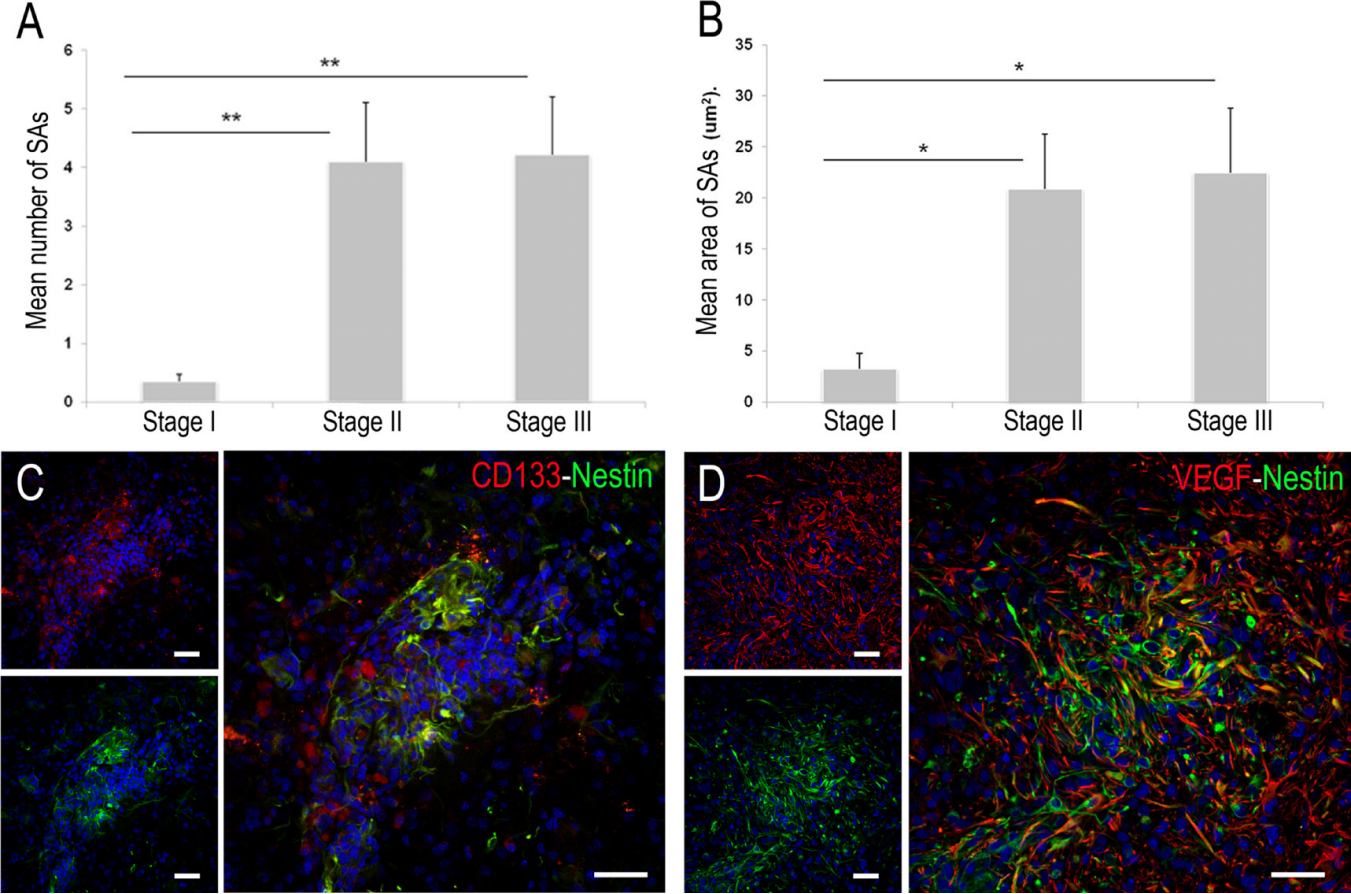
Quantitative and inmunofluorescence study of spheroid-like aggregates (SAs) (**A**–**B**) Graphics show the enhancement of their density and size during the ENU-glioma progression. There is no significant difference between stage II and III. ^*^p < 0.05, ^**^p < 0.01, ^***^p < 0.001. (**C**) SAs showing a high density of small round neoplastic cells. Some of them express nestin (green), CD133 (red) and nestin/CD133 (yellow). (**D**) VEGF stain is mainly shown in astrocytes located externally to SAs. Few nestin+ cell processes share VEGF positivity (yellow). Scale bar of 50 μm.

SAs are groups of at least six nestin+ neoplastic cells with a small round morphology and no cell processes. They predominate in stages II and III and their density and size increase according to tumour malignancy (Figure 2A, 2B). The density and size of SAs in neoplasia is several fold higher in stage III than in stage I (mean number of SAs = 4.2 ± 0.9, 0.36 ± 0.1 and mean size = 22.4 ± 6.3, 3.3 ± 1.5 µm^2^, respectively). The significant increase is found in stage II with respect to stage I (*p <* 0.05). Distribution of stemness markers in SAs also varies according to glioma malignancy. The smallest SAs (stage I) show a few nestin+ cells. More neoplastic cells stained by nestin and a few cells positive for CD133 are found in SAs from stage II. At this intermediate stage, nestin staining is also shown in large cells with cell processes. However, CD133 immunoexpression is only observed in the soma of small cells. The largest SAs (stage III) display different populations of cells: small round cells that express nestin, CD133 and nestin/CD133 (Figure 2C) and numerous GFAP+ astrocytes with long processes surrounding SAs. The majority of the astrocytes co-localize with VEGF. Indeed, a few of the astrocyte processes are marked with nestin (Figure 2D).

### Hypoxic cell distribution related to CD133 expression

**Figure 3:**
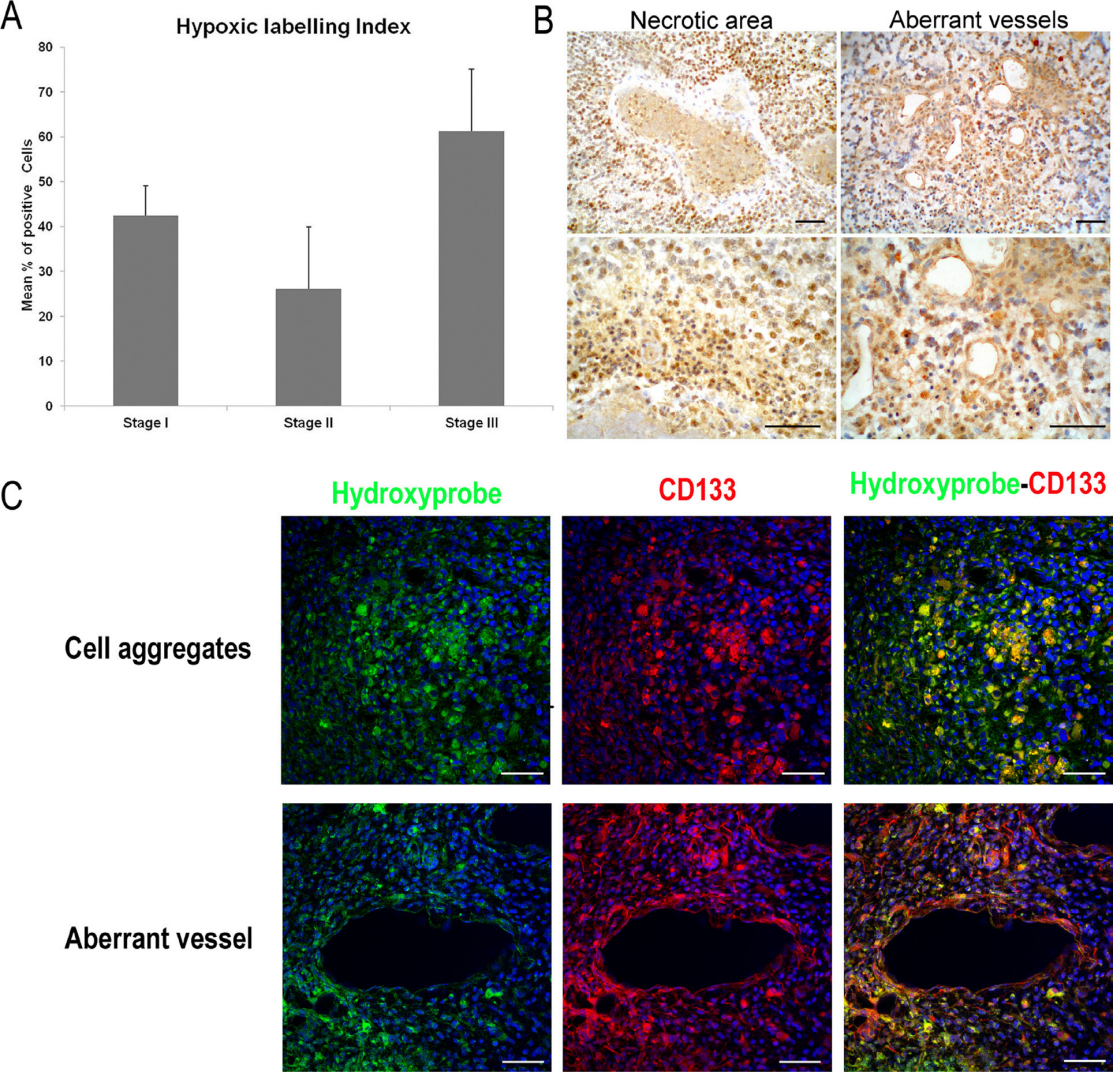
Detection of tissue hypoxia in ENU-glioma by Pimonidazole hydrochloride (Hydroxyprobe™-1 solution; Chemicon) (**A**) Graphic representation of hypoxic cell density in three stages of ENU-glioma development. ^*^p < 0.05, ^**^p < 0.01, ^***^p < 0.001. (**B**) Hypoxic cell distribution in stage III shown by DAB, in perinecrotic palisade and surrounding the endothelium of dilated vessels. (**C**) Confocal immunofluorescence images showing hypoxyprobe (green) and CD133 (red) staining identifying the presence of GSLCs in hypoxic tumour areas of stage III. Numerous hypoxic cells co-express with CD133 (yellow), forming aggregates of neoplastic cells within the tumour and the perivascular area of aberrant vessels. Scale bar of 50 μm.

Quantitative results from the “hidroxyprobe” assay show a decrease of hypoxic cell density between stages I-II and a great increase from II to III (Figure 3A). Although stage III presents the highest density of hypoxic cells (Hypoxic-LI of 61.3 ± 13.8%), no differences among groups are found. Morphologically, positive scattered neoplastic cells are distributed throughout the proliferative mass in the initial stage (I), while in the most malignant stage (III), numerous strongly positive cells are accumulated in hypoxic areas such as pseudopalisading necrosis or around aberrant microvessels (Figure 3B). In stages II and III some of these hypoxic cells show CD133 expression as well (Figure 3C).

### Notch-1+ undefined cells in advanced ENU-glioma stages

**Figure 4:**
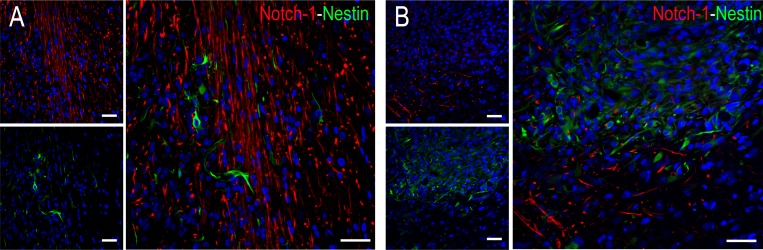
Co-expression study of Notch-1 (red) and nestin (green) in stage III (**A**) Distribution of Notch-1+ cells with fusiform morphology detected in the peripheral area of the tumour. (**B**) Notch-1+ elongated cell processes of undefined cells around SAs. There is no co-expression with nestin antibody. Scale bar of 50 μm.

Notch-1 immunopositivity is observed in cell processes of undefined cells distributed in the tumour border and around large SAs of stages II–III. Notch-1 co-expression with other antibodies used in this work is not detected (Figure 4).

### OPN overexpression in the border area of stage III

OPN immunoexpression is found in all glioma stages. There is an increase in OPN-LI according to malignancy, being statistically significant between stages I–III and II–III (*p <* 0.001 and *p <* 0.05, respectively). Distribution of OPN+ cells within gliomas displays a few small OPN+ cells (OPN-LI of 4.5 ± 1.8%) dispersed inside the proliferative mass in stage I, while OPN+ large cells in stages II and III (OPN-LI of 7.7 ± 0.8% and 12.3 ± 1.2%, respectively) predominate on the border.

**Figure 5:**
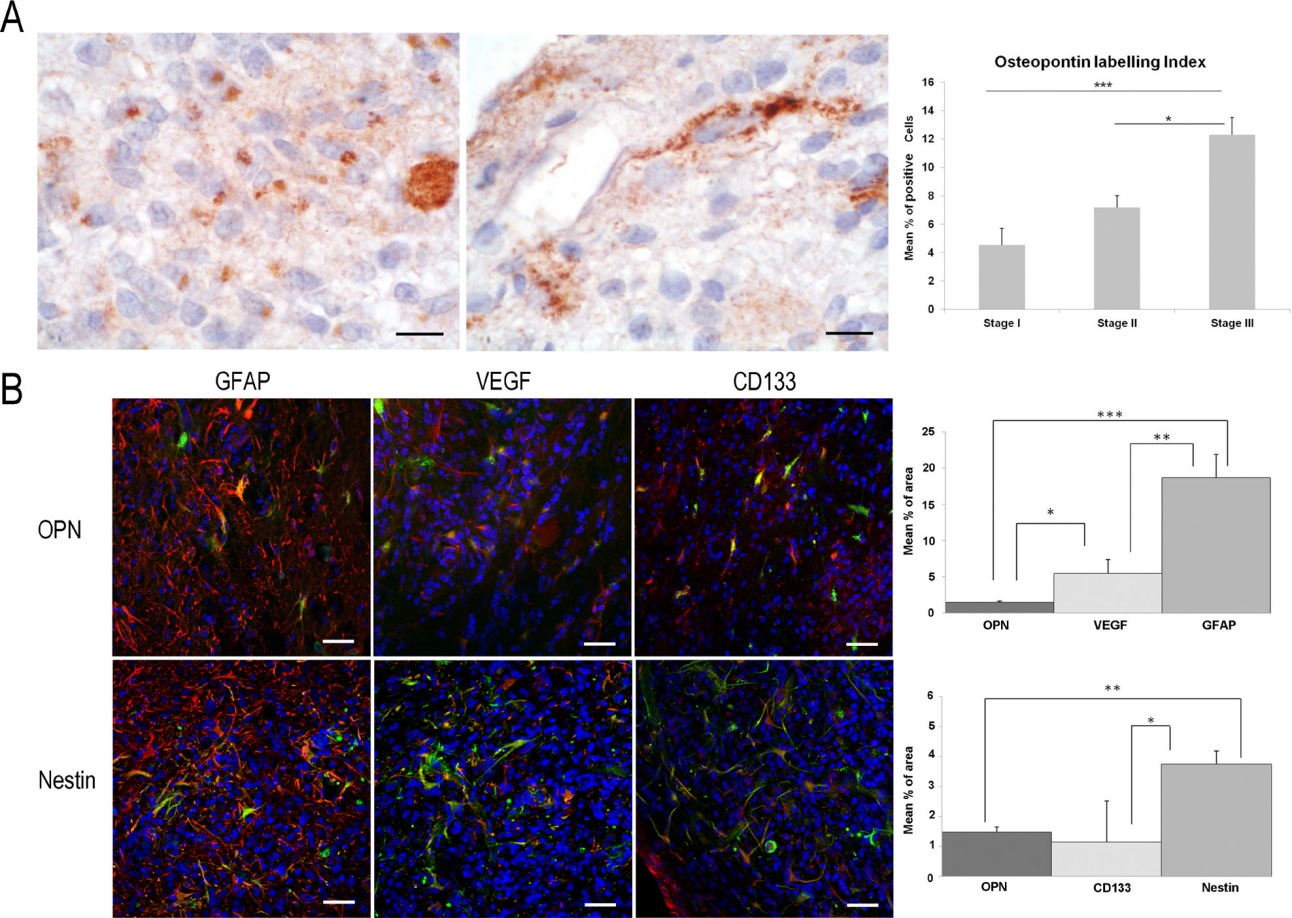
Study of OPN immunoexpression in ENU-glioma stages of development There is an enhancement of the density of OPN+ cells corresponding to ENU-glioma malignant process. (**A**) In advanced stages II–III, these cells present different morphologies, as shown by DAB. Small round shape cells, cells of large soma and elongated cells contacting with a tumour microvessel is found within the neoplasia. (**B**) In the periphery area of stage III, many astrocyte-like cells tend to cluster. Confocal images of double immunofluorescence show positivity of astrocyte-like cell for OPN, nestin (green); VEGF, GFAP, and CD133 (red) (Hoechst counterstained, blue). Some of OPN+ cells and some of nestin+ cells co-express with GFAP, VEGF and CD133 (yellow). OPN expression is similar to CD133 but significantly lower than GFAP, VEGF and nestin one. Nestin is significantly lower than GFAP but similar to VEGF. Graphics show the osteopontin labelling index as mean percentage of positive cells (A) and quantitative study of the immunoexpression of the studied antibodies as mean percentage of border surface occupied by positive cells (B) ± typical error. ^*^p < 0.05, ^**^p < 0.01, ^***^p < 0.001. Bar scale of 10 μm (A) and 50 μm (B).

Stage III shows some OPN+ small cells with scarce cytoplasm and no cellular processes inside the tumour, coexisting with some OPN+ cells with large cytoplasm and numerous cell processes as well as some large cells distributed adjacent to the microvasculature (Figure 5A). In the border area, there are abundant GFAP+ astrocyte-like cells occupying the 18.68 ± 3.22% of total surface. A few of them co-localize with OPN, VEGF and OPN/VEGF. Immunoexpression of OPN is statistically less than VEGF (1.4 ± 0.17% and 5.48 ± 1.9%, *p* = 0.011) (Figure 5B). Nestin and CD133 immunoexpression is also found in this area, being nestin expression significantly higher than that of CD133 (*p* = 0.042). Moreover, quantitative study reveals no statistical differences between nestin and VEGF expression, nor between OPN and CD133 expression. Most nestin+ cells co-express VEGF and most OPN positive cells co-express CD133 (Figure 5B).

## DISCUSSION

Previous studies have suggested that GSLCs are involved in angiogenesis and glioma progression [2, 7, 35]. Thus, understanding the mechanisms underlying the maintenance of GSLCs is crucial for the development of antiangiogenic therapies. In this study, we investigate the association between two molecules involved in glioma neoangiogenesis, OPN and Notch-1, and two stem cell markers, nestin and CD133.

In ENU-gliomas, in a similar way to human brain tumors, histological heterogeneity with hierarchical differentiation potential is shown [36]. Various cell populations have different capacities for tumor growth depending on their undifferentiating status [37]. The immunostaining against Nestin or CD133 allows us to identify a subgroup of small round cells with no processes, suggesting they are in a stemness state or phase, and another subgroup of differentiated large cells with numerous processes [38].

The expression of both markers increases during the development of ENU-induced gliomas, corroborating their prognostic significance [39]. Immunopositive cells for CD133 appear in more advanced stages than nestin ones do. Moreover, results reveal that cells co-expressing nestin/CD133 tend to aggregate in hypoxic niches. Some authors state that interconversion between non-CSCs and CSCs could be shifted in one way or another in response to specific microenvironmental factors, such as hypoxia, acidic stress, and metabolic stress [40]. In various tumors, hypoxia influences the dedifferentiation of cells and acquisition of nestin/CD133 positivity [27, 41].

### Notch-1 and spheroid-like aggregates

Before angiogenesis starts, neoplastic cells have to acquire a determined phenotype [42]. In addition, the tumoural microvasculature experiments changes related to the relative tissue hypoxia taking place, which leads to distorted microvessels, irregular regional blood flow and disturbed Blood-Brain Barrier function. Consequently, intratumoral blood perfusion is altered and tissue hypoxia increases [29, 34].

Hypoxia induces Notch signalling, which in turn promotes and reprograms malignant cells towards GSLC phenotype [43]. Hypoxic pre-conditioning status could be mediated by HIF-1alpha and Notch-1 pathway [26, 44]. In advanced stages of ENU-glioma (II, III), hypoxic cells are found inside pseudopalisading necrosis and around bulk aberrant microvessels. These abnormal vascular niches mimic the normal neural stem-cell niches and promote proliferation of the GSLCs, housing and protecting them [45]. Thus, next to and even around these vessels, SAs are found [30].

SAs resemble the *in vitro* tumour spheres where different cell populations co-exist. In our previous work, we described Nestin+, CD133+, CD133/nestin+ cells and neighbouring supportive cells like astrocytes expressing GFAP and VEGF [30]. Now, we can include overexpression of Notch-1 in cells adjacent to these structures. Notch-1 and VEGF may be microvascular niche-derived factors that actively maintain the stem-like state and promote proliferation [45, 46]. Similar cell aggregates have been previously described using histochemistry for butyrylcholinesterase (BChE) [29]. This choline esterase has a very relevant function in neurobiology as a growth factor, being involved in cell proliferation and differentiation [47].

Notch-1 and VEGF are overexpressed in ENU-glioma intermediate stage (II), corresponding to angiogenesis switch; in parallel, there is a significant increase in size and density of SAs [2, 30]. These findings could explain the role of Notch-1 and its ligands in glioma survival and proliferation mediated by stem-like cells [14]. Moreover, it is known that expression of Notch-1 and VEGF is induced by hypoxia. Therefore, hypoxia is crucial for the survival of stem-like cells and modulation of these niche microenvironment factors [48]. Stem-like cells and SAs are adaptive changes in order to survive in adverse microenvironmental conditions.

### OPN in the neoangiogenic area of ENU-glioblastoma

OPN has been widely associated with tumour progression, development of metastasis and characteristic resistance to treatment of cancer stem cells [49]. This glycoprotein has been identified as a prognostic factor in human GBM [50]. We corroborate this property in the ENU-glioma model, finding an early onset of OPN expression in a few cells displayed in stage I, which increases during neoplastic development, and tends to cluster in the border of the glioma, reaching its highest expression in stage III.

The peripheral area of ENU-GBM (stage III) was characterized as a neoangiogenic border because of VEGF overexpression and microvascular proliferation [30, 33]. Aggregates of some nestin+, CD133+ and nestin/CD133+ elongated cells associated with endothelial cells were described in this area [29, 34]. In common with some other authors, we proposed that these nestin+ and CD133+ cells may be selected cells with invasiveness and proliferative capacity which use the extracellular matrix of the vessel wall to migrate and infiltrate the brain parenchyma [51, 52]. Now, in the present work, we describe numerous astrocyte-like cells near to glomeruloid vessels that overexpress OPN and also the stemness markers nestin and CD133. It could be another fraction of nestin/CD133+ neoplastic cells, already described by Wang *et al.* (2010), related to endothelial differentiation, and not with a migratory or invasive role [51]. They could be involved in the angiogenesis process, generating tumor vessel prolongation [52]. Moreover, in the neoangiogenic area of ENU-GBM, some astrocyte-like cells co-express OPN/VEGF. It has been reported that OPN acts synergically with VEGF, as they induce the expression of one another in tumor cells [53]. Experimental evidence suggests that OPN may affect angiogenesis by endothelial cells directly via PI3K/AKT- and ERK-mediated pathways with VEGF acting as a positive feedback signal [54]. In the ENU-glioma model, we have shown a close relationship between tumor microvascular endothelium, eNOS, VEGF and the angiogenesis process [55]. Adding to the above, this recent study describes expression of OPN in cells attached to the tumor microvascular network. Considering this, we agree with Wang *et al.* (2011) that upregulation of OPN in glioma cells could stimulate formation of vascular endothelial cells via activation of VEGF and the avβ3/PI3-K/AKT/eNOS/NO-dependent signaling pathway [56].

In summary, our findings reveal that Notch-1 and OPN are overexpressed in the intermediate (II) and advanced (III) stages of ENU-gliomas. Both molecules have a relationship with VEGF. In these stages, there is a significant increase of cell aggregates composed of populations of cells expressing nestin+, CD133+ or nestin/CD133+ simultaneously. Notch-1 and VEGF are distributed around these cell aggregates. Thus, they would actively contribute to maintaining the stem-like state and proliferation capacity of GSLCs [57, 58]. On the other hand, we also demonstrate that OPN and VEGF are overexpressed in the neoangiogenic border, where the area immunopositive for VEGF is significantly higher than that for OPN. Moreover, the immunopositive area for OPN is very similar to that positive for CD133, and the VEGF-positive area is similar to the nestin one. Some areas display co-expression of OPN/CD133. These findings corroborate that OPN and VEGF are involved in glioma progression mediated by GSLCs [53, 54, 56, 58]. That supports the interest in GSLCs, OPN and Notch-1 as possible targets for the treatment of GBM. Intermediate stage ENU-glioma could be a good tool to develop new antitumour therapeutic strategies.

## MATERIALS AND METHODS

### Experimental animal tumor model and hydroxyprobe assay

6 Sprague-Dawley adult rats were intraperitoneally injected with a single dose of N-ethyl-N-nitrosourea (ENU, 80 mg/kg.b.w, 10 mg/ml in 0.9% NaCl; E2129, Sigma-Aldrich, Spain) at 15th day of pregnancy. Offspring rats were analyzed every two days from 4th to 10th of rat age to establish their health status and to identify neurological clinical sings. The health status was recorded taking into account abnormal postures, abnormal vocalizations, unusual weight loss, and extreme immobility or swallow eyes. Rats were asymptomatic until 6 month of age. The most relevant neurological signs recorded were: lateralization of the head, ataxia, anxiety and increase of rearing. Symptoms progressed with the rat age and they could come to show severe symptoms, such as numbness, apathy, seizures and death.

In line with our previous work, to detect the glioma neoplasia, offspring rats were sacrificed monthly between 6 to 10 months old. Fifty-three of these rats that displayed glioma were selected for this study. A quarter of the animals display a multifocal location of neoplasia.

Four hours prior to euthanasia, 11 animals were intraperitoneal injected with 60 μg/kg.b.w. of Pimonidazole hydrochloride (60 μg/mg, Hydroxyprobe™-1 solution; Chemicon).

All experimental methods were carried out following the Spanish Royal Decree 1201/2005 BOE published October 21st 2005, and the 2003/65/CE from the European Parliament and Council of July 2003. The Ethical Committee for Animal Welfare (CEBA) of the University of the Basque Country, Leioa, Spain (CEBA/154/2010/) approved the protocols.

### ENU-glioma screening

Gliomas were *in vivo* identified by magnetic resonance imaging (MRI, Biospec BMT 47/40, Bruker, Ettlingen, Germany). To aid in tumor visualization, animals were injected i.p. with 1.5 ml/kg b.w. of gadolinium (Gd-DTPA) (Magnevist, Schering).

Then rats were transcardially perfused with 2% PFA, their brains were removed and immersed in the same solution at 4° C overnight. Tumors were detected by stereoscopic microscopy (Lan Optics). Some coronal sections including the tumor were embedded in paraffin wax (Histosec^®^ Merck) and others were stored in 30% sucrose until the tissues sink.

### Evaluation of tumour size and histologic analysis

For the histopathological study, hematoxylin-eosin (H&E) staining was carried out on 4 mm sections. Sections were observed for the presence of histological features, such as cellular anaplasia, atypical mitosis, microvascular proliferation, hemorrhages, necrosis or cysts. The tumor area was measured using a reticule of 62,500 μm^2^ (at × 40 magnification) as our previous work [33, 34].

### Immunohistochemistry

Immunohistochemical assay was carried out on 4 μm sections against nestin (mouse monoclonal, 1:200; sc-33677, Santa Cruz, CA), osteopontin (OPN, mouse monoclonal, 1:200; sc-21742, Santa Cruz, CA) and Ki-67/MIB-5 (Clone MIB-5; mouse monoclonal, 1:100; M7248, Dako, Denmark) using the conventional ABC method (Elite ABC Kit, Vector Laboratories, Burlingame, CA). In the case of the Pimonidazole hydrochloride, the manufacturer’s protocol was followed (Hydroxyprobe™-1 solution; Chemicon, USA). The reaction product was developed by 3.3-diamino-benzidine (DAB, 0.25 mg/ml; 8001, Sigma-Aldrich, Spain) and H_2_O_2_ (0.01%) followed by haematoxylin counterstaining. Sections were finally dehydrated and covered with DPX mounting medium (Sigma-Aldrich, Spain). Negative controls in which the primary antiserum was omitted were also included in each staining run.

To assess the co-expression of the different markers, double staining was carried out in free-floating sections. Briefly, 40 μm free-floating sections were incubated with blocking solution (10% BSA, 3% Triton X-100 in 0.1 M PBS) for 2 h. Then they were incubated overnight at room temperature with a cocktail of primary antibodies in 1% BSA containing 0.1% Triton X-100. Primary monoclonal antibodies used were nestin (1:400; sc-21247, Santa Cruz, CA) and osteopontin (OPN, 1:300; sc-21742, Santa Cruz, CA). Polyclonal antibodies are vascular endothelial growth factor (VEGF, 1:200; sc-152, Santa Cruz, CA), GFAP (1:400; sc-6170, Santa Cruz, CA), CD133 (1:100; ab-16518 Abcam, Cambridge, UK), Glucose transporter 1 (GluT-1, 1:200; 07-1401, Merck Millipore, Germany) and Notch-1 (1:200; bs-1335R Bioss Antibodies Inc., USA). Subsequently, sections were incubated for 2 h with a cocktail of secondary fluorescent antibodies: Alexa Fluor 568 and 488 (1:400, Invitrogen, Spain). Then, sections were incubated with Hoechst solution for 10 min. Finally, sections were rinsed, mounted on gelatin-coated slides and cover-slipped in an aqueous medium (Vectashield Mounting Medium H-100). Primary antibodies were omitted for control samples. Images were acquired with Olympus Fluoview FV 500 confocal fluorescence microscopy using sequential acquisition to avoid overlapping of fluorescence emission spectra. The images were treated with *FV 10-ASW 1.6 Viewer* and *Adobe Creative Suite* 4.

### Quantitative studies

Mean number of positive cells for nestin (nestin-LI, labelling index), osteopontin (OPN-LI) and Ki-67 (PI, tumour proliferation index) were calculated in 81 gliomas and mean density of hypoxic cells (Hypoxic-LI) in 11 gliomas. To calculate these indexes, we followed the protocol described by Bulnes & Lafuente [30, 33]. 400 tumour cell nuclei were counted at high magnification (×400) from the more representative area of the tumour, and the percentage of cells expressing the antigen in any way was reported. The quantitative evaluation was performed by the same researcher to avoid inter-observer variability. Only positive cells not adhered to vessels or cells are individually localized not forming aggregates were counted.

Measurements of SAs (aggregation of 6 or more nestin+ cells), were performed in sections from 81 gliomas following our previous work [34]. The parameters of SAs analyzed were: incidence (% of tumors displaying one or more SAs), density (mean number of SAs per mm^2^ of tumor) and size (mean area occupied by the SAs, expressed in μm^2^). The measurements were carried out using *Image J* program (1.48 n) and the mean value per ENU-glioma stage was calculated.

Immunoexpression of OPN, VEGF, GFAP, nestin and CD133 was analysed in confocal photomicrographs (pixel size of 32181 μm^2^) from 7 samples of the border area of stage III. The area occupied by the cells stained by these antibodies was measured using *Fiji-win64* program. Values were expressed as the percentage of area stained in 101249 μm^2^ of tumour border.

### Statistical analyses

All statistical data were analyzed using SPSS statistical software (version 23.0 from IBM, Spain). Prior to analysis, data was examined for normal distribution using the Kolmogorov-Smirnov test and for homogeneity of variances using Levene’s test. To compare the parameters studied in the three different ENU-glioma stages we used two-way ANOVA with posthoc analysis (posthoc tests use the Bonferroni correction for equal variances or Tamhane’s T2 correction for unequal variances). Data was described as mean ± typical error. Significance was declared at *p <* 0.05.

## Abbreviations

OPN: osteopontin
GSLC: glioma stem-like cell
SAs: spheroid-like aggregates
ENU: N-ethyl-N-nitrosourea
GBM: glioblastoma
CNS: central nervous system
VEGF: vascular endothelial growth factor
EPO: erythropoietin
eNOS: endothelial nitric oxide synthase
HIF-1α: hypoxia inducible factor 1 alpha
SIBLINGs: Small Integrin-Binding Ligand N-linked Glycoproteins
MRI: magnetic resonance imaging
DAB: 3.3-diamino-benzidine
BChE: butyrylcholinesterase
CSC: cancer stem cells
Nestin-LI: nestin labelling index
Ki-67 PI: tumour proliferation index
OPN-LI: osteopontin labelling index
Hypoxic-LI: Hypoxic labelling index
GluT-1: glucose transporter 1
